# High BMI Predicts Attention to Less Healthy Product Sets: Can a Prompt Lead to Consideration of Healthier Sets of Products?

**DOI:** 10.3390/nu13082620

**Published:** 2021-07-29

**Authors:** Christopher R. Gustafson, Kristina Arslain, Devin J. Rose

**Affiliations:** 1 Department of Agricultural Economics, University of Nebraska-Lincoln, Lincoln, NE 68583, USA; 2 Department of Food Science and Technology, University of Nebraska-Lincoln, Lincoln, NE 68588, USA; kristinaarslain@gmail.com (K.A.); drose3@unl.edu (D.J.R.); 3 Department of Agronomy and Horticulture, University of Nebraska-Lincoln, Lincoln, NE 68588, USA

**Keywords:** consideration set, attention, weight status, health prompt, food choice, experiment, online supermarket

## Abstract

While the food environment has been implicated in diet-related health disparities, individuals’ ability to shape the food environment by limiting attention to a subset of products has not been studied. We examine the relationship between BMI category and consideration set—the products the individual considers before making a final choice—in an online hypothetical shopping experiment. Specifically, we focus on the healthiness of the consideration set the individual selected. Secondly, we examined the interaction of a health prompt (versus a no-prompt control) with BMI category on the healthiness of the consideration set. We used linear probability models to document the relationship between weight status and consideration set, between prompt and consideration set, and the effect of the interaction between prompt and weight status on consideration set. We found that (1) obese individuals are 10% less likely to shop from a consideration set that includes the healthy options, (2) viewing the prompt increased the probability of choosing a healthy consideration set by 9%, and (3) exposure to the prompt affected individuals in different BMI categories equally. While obese individuals are more likely to ignore healthier product options, a health-focused prompt increases consideration of healthy options across all BMI categories.

## 1. Introduction

The prevalence of overweight and obesity in the adult US population is currently over 70%, which has led to a crippling disease burden related to high body mass index (BMI) [1]. High BMI has been identified as a causative factor for the decrease in life expectancy in the US over the past few years [2,3]. Being overweight or obese is linked to poorer health outcomes, including a higher probability of developing non-communicable diseases, such as type 2 diabetes, cancer, and heart disease [4]. Overweight and obesity has become a leading cause of death in the US [5], and has contributed to millions of deaths globally [1]. Additionally, it is estimated to cost the US USD 150 billion per year in direct costs (in 2008 dollars), and USD 3–6 billion annually in indirect costs [6,7,8]. While genetics, physical activity, and other factors contribute to high BMI, diet is consistently recognized as a key behavioral element contributing to the high rates of overweight and obesity in the US [9]. According to recent research on energy expenditure, diet, and weight across hunter-gatherer and sedentary populations of the same ethnic groups, diet, rather than physical activity, appears to be the decisive factor in promoting higher BMI [10,11,12]. A diet composed of highly processed, calorically dense foods and low in fruits and vegetables leads to weight gain [13].

One area of research on diet and obesity has focused on the impact of food environment on the nutritional quality of individuals’ diets. A significant amount of this research has examined food deserts—areas that are typically in highly urban or rural settings in which residents lack ready access to healthy foods [14]. Links identified between food deserts and higher rates of overweight and obesity [15] have inspired public and private investment to eliminate food deserts [16]. However, while eliminating food deserts causes people to feel they have greater access to healthy foods, the quality of their diets does not improve [17,18,19]. This suggests that food deserts may actually reflect average local consumer demand for healthier versus less healthy foods, and that higher average BMIs in those areas are not caused by the environment but, like the food environment, reflect individuals’ food preferences [20].

The food environment as experienced by shoppers can vary, even within a single retail outlet, because people direct their attention to products differently. Shoppers face a vast array of products and product categories in food retail outlets; a typical well-stocked supermarket has tens of thousands of products, with many individual product categories containing hundreds of unique products [21,22]. With such a large number of products to select from, consumers cannot consider all available products. Instead, consumers form a “consideration set”—a small set of alternatives that the individual considers and ultimately chooses from [23,24,25]. In a recent eye-tracking study of supermarket shoppers, 67% of purchased products were chosen without the shopper considering any other products in that category [26]. The formation of consideration sets reflects people’s preferences [27], but is also influenced by what people expect the benefit of expanding their consideration set will be [28]. For example, in the context of food and health, there is significant evidence that people associate healthier foods with higher prices [29,30,31]. This assumption may deter them from considering healthier options when, in fact, the relationship between health and price is not definitive [32].

Research suggests that attention to nutrition information and health messaging is important in promoting healthy choices [33,34,35,36,37]. Intuitively, consideration of healthy products when making a purchase decision is also important, and is a necessary precondition to the purchase of healthy foods. However, there is little evidence that documents how individuals attend to a large array of product options when they have the ability to purposefully direct their attention. They may choose to consider all available products, or may restrict themselves to a small subset of options.

While individuals’ decisions about which products to consider are overlooked in most studies examining the healthiness of food choices, they shape the products and product information that people encounter when making choices. In this study, we examine the sets of products that individuals in an online supermarket pay attention to when making a food choice as a function of BMI. We focus, in particular, on how the weight status of individuals predicts their consideration of products. We then examine the effects of exposure to a fiber-based point-of-decision prompt (PDP) on consideration set, in order to evaluate whether prompts affect behavior differently for individuals of differing weight status.

## 2. Materials and Methods

### 2.1. Survey Design

#### 2.1.1. Limited Product Consideration and Attention to Product Information

The design of our experiment aimed to (1) document choice process variables—such as participants’ considerations sets and the information that they used—that contributed to their ultimate product choice, and to (2) examine the effects of a fiber-based prompt message on the choice process and product variables. We were interested in examining how these relationships differed between groups of individuals with different body weight status in order to establish whether differences in attention to products and information may reinforce body weight status. We developed an online food choice experiment that was structured to replicate features common to online supermarket shopping interfaces.

There were two primary stages to the data collection process: (1) a shopping task, and (2) a survey. In the first stage, research participants faced three product categories: cereals, breads, and crackers. Participants first made a decision about the set of products to view in each category. Participants could examine all product options (*N* = 33 for each food category), or they could select to view a subset of products (*N* = 11 per subset). This breakdown reflects design features in many physical stores and online shopping environments, which permit consumers to quickly narrow the total set of products to a preferred subset. While ultimately structured to reflect real-world retail design, the subsets additionally separated products into less healthy, moderately healthy, and healthy options based on the Guiding Stars nutritional rating system rubric (https://www.guidingstars.com, (accessed on 12 March 2020).

In the Guiding Stars system, products are graded based on nutrient content on a 0–11-point scale. Products receive points if they meet or exceed criteria for vitamins, minerals, fiber, whole grains, and omega-3 fatty acids, while they lose points if they exceed benchmark levels of saturated fat, trans fat, added sodium, added sugar, and artificial colors (per standardized 100-calorie portion). Points are converted into stars—which constitute the consumer-facing information—in the following way: products with 0 points from the rubric receive zero stars; 1–2 points receive one star; 3–4 points receive two stars; and 5–11 points receive three stars. We created balanced product subsets, in which products received zero (11 products), one (11 products), and two or three stars (11 products) in each product category. We combined two- and three-star-rated products into one category because it was difficult to find enough products receiving three stars to create a separate category.

The subsets were described to participants according to examples of the products they contained, in order to avoid priming participants to explicitly think about the products in terms of health (again, following the design of real-world retail sites). In our experiment, the cereal sets were labeled as “Cereals such as Frosted Flakes, Froot Loops, Reese’s Puffs”, “Cereals such as Corn Flakes, Crispix, Special K”, “Cereals such as Cheerios, Wheat Chex, Grape Nuts”, and “All options”. Bread and cracker subsets were presented in the same manner.

The participants’ choices of product set determined the products viewed by the participants. After viewing the available products in a category, the participant then selected a product to “purchase”. The participant could also indicate that they would not purchase any of the available items (3% of participants indicated that they would not purchase a product in at least one of the three product categories). This option—indicating that they would not purchase any of the items—was always listed as the last option, while the presentation of the other available products was randomized. The product options were presented in a three-column format, with a photograph and the name of each product presented prominently. Underneath each product, the nutrient contents per serving for calories, fiber, fat, sodium, and sugar, as well as the price, were listed. After making choices in all three product categories, participants answered survey questions about their choices, typical shopping practices, and demographics.

The products included in the experiment were real brands that are widely available at regional and national supermarket chains in the US. These products were selected to represent a range of taste and nutrient profiles. Store brands were excluded to avoid differences in regional familiarity with products. The specific products included in the three categories are presented in Table A1, Table A2 and Table A3 in Appendix A. Each product also had a price associated with it, which was based on retail prices at the time at which the survey was conducted. We included a message in the introductory materials for the shopping task experiment encouraging participants to imagine they were making real choices with real money, which has been found to reduce hypothetical biases in economic choices [38].

For this paper, the important questions included in the post-experiment survey included questions about self-reported height and weight, and demographic variables, such as gender, age, income, and education. We used participants’ self-reported height and weight data to calculate each individual’s BMI, and then created a category variable based on BMI. Individuals with a BMI < 25 were categorized as normal weight; individuals were categorized as overweight if 25 ≤ BMI < 30; individuals with a BMI > 30 were categorized as obese. The dependent variable in the analyses was created by indicating whether the participant chose a consideration set that included the healthiest items (those that received a rating of 2 or 3 Guiding Stars). The healthy subset and the set that included all available options for each product type both qualified based on this definition. The “healthy consideration set” was coded as 1 for respondents that chose to view the healthy subset or the “all options” product set for a particular product type, and a 0 otherwise. We averaged the number of healthy consideration sets each participant chose to view across the three product categories to create the dependent variable used in the analyses.

The experiment and survey vehicle was programmed in Qualtrics XM (2021, SAP, Provo, UT, USA). The survey was distributed to adults 19 years of age or older in the United States through Amazon Mechanical Turk from 15 April to 20 April 2020. No additional inclusion or exclusion criteria were used. The University of Nebraska-Lincoln IRB approved the research (IRB protocol #20201020721EX). All participants provided informed consent before participating in the research.

#### 2.1.2. Effects of Exposure to a Fiber Information Prompt on Individuals of Differing Weight Status

To examine how individuals of differing weight status reacted to a fiber information prompt, we randomized participants into control and one of two prompt conditions. The prompt messages varied only in their inclusion/exclusion of second-person pronouns, and did not result in differences in choice behavior [36], so we aggregated them into one condition. The prompt presented information about the health benefits of fiber, comprising weight management, reduction of disease risk, lowering of cholesterol, regulation of the digestive system, blood sugar control, and effects on the gut microbiome. Participants in the PDP condition viewed the PDP just before beginning the shopping task, while control group participants immediately began the shopping task.

### 2.2. Survey Analysis

We analyzed data using the open-source statistical analysis software, R [39]. We report summary statistics and use multivariate linear regression to analyze the data (and report ordinal regression results in Appendix B, Table A4). The dependent variable in the analysis was the proportion of times a participant chose a consideration set that included the healthiest options (either the healthiest subset itself, or the set that contained all available products). This variable ranged from 0 to 1, calculated by taking the total number of times the individual chose a healthy consideration set divided by the three product categories for which individuals made consideration set decisions. We report the results of a multivariate linear probability model because this model provides estimated coefficients that are directly interpretable as probabilities [40]. In the linear probability model, interaction terms capture differential responses to the prompt by individuals of differing weight status. We report the results of multivariate ordinal logistic regression models in Appendix B, which mirror the linear results in significance and direction.

We report results from six regression models. We first examined the relationship between BMI category and consideration set in order to evaluate whether there were significant relationships between participants’ BMI status and the sets of products they choose to pay attention to. Next, we added an indicator variable capturing whether a participant was exposed to the prompt (Prompt) to examine the impact of the prompt on the probability of examining a consideration set with the healthiest products. Finally, we examined the interaction of BMI category variables with the prompt, to study whether the people in different BMI categories responded differently to the prompt. We report each of these models with and without common demographic variables (gender, age, income, and education) to check the robustness of results to the inclusion of these variables. The inclusion of demographic variables did not affect the estimates of the target independent variables but did require more participants to be dropped from the dataset because of “prefer not to answer” responses, so the number of observations varied slightly between regressions that did and did not include demographic variables. We considered *p* < 0.05 to be statistically significant.

## 3. Results

Summary statistics for demographic and weight status variables are presented in Table 1. There were no significant differences between demographic and weight variables in the control and prompt conditions (using a chi-squared test to test for differences in the distribution of females across conditions, and *t*-tests to examine differences in age, household income, education, and BMI). Approximately 36% of participants were female. The average age of participants was 37 years, and their mean household income was approximately USD 60,000. Participants had received around 16 years of education (approximately equivalent to a bachelor’s degree). The average BMI of participants was 25.5, which is in the “overweight” category.

As we were interested in the relationship between individuals’ weight status and the sets of food items they chose to consider, we examined the distribution of respondents across BMI categories (i.e., normal weight, overweight, and obese) for the control and prompt conditions (Table 2). The distribution of participants among BMI categories was not significantly different between the control and prompt conditions (Chi-squared = 0.387; df = 2; *p* = 0.82). In both conditions, slightly over 40% of participants were in the normal weight category, slightly over 20% were overweight, and around 35% were obese.

The results of the six regressions examining the relationship between BMI category and consideration set are presented in Table 3. In Regression 1, we found that individuals with BMIs in the obese category were 10% less likely (*p* < 0.001) to select a consideration set that contained the healthier product options than normal-weight individuals (the omitted weight category in the regression). This result did not change when we included demographic control variables (Regression 2). Individuals who fell into the overweight category did not behave significantly differently from normal-weight individuals.

In Regressions 3 and 4, we included a variable that captured the effects of participants being exposed to the prompt message (along with controlling for demographic variables in Regression 4). Again, we found that obese individuals were 10% less likely to choose a consideration set that contained the healthiest items (*p* < 0.001) in both regressions. We also found a statistically significant, positive effect of the prompt on the probability of selecting a healthy consideration set. In both regressions, we found that exposure to the prompt increased the probability that an individual would select a consideration set that contained the healthier items by 9% (*p* < 0.001).

In Regressions 5 and 6, we introduced interaction terms between exposure to the prompt and weight status, in order to examine whether individuals of varying weight status responded differently to the prompt message. We continued to find that obese individuals were around 10% less likely to select a healthy consideration set (*p* < 0.05), corroborating the results of Regressions 1–4. We found an effect of the prompt that was consistent with the findings in Regressions 3 and 4: exposure to the prompt increased the probability that participants selected healthier consideration sets by 9% (*p* < 0.05). Furthermore, we did not find statistically significant interactions between weight status and exposure to the prompt. The point estimates of the interaction terms between overweight weight status and the prompt and obese weight status and the prompt were both small—between 1 and 3% in absolute value—and not statistically significant.

## 4. Discussion

Recent work attempting to untangle the relationship between the food environment and higher average BMIs has demonstrated that eliminating a food desert does not consistently lead to healthier food purchases [17,18,41,42]. While moving from a high-obesity area to a low-obesity area does improve the nutritional quality of a household’s food purchases over time, the effect is relatively small [43], suggesting that demand-side factors play an important role in explaining the lack of healthy food options [20]. In a more controlled setting, laboratory research has documented attentional biases of individuals with high BMI to more indulgent foods and has shown that overweight/obese individuals are willing to pay more for these foods [44,45,46,47,48]. However, to the best of our knowledge, this is the first work that documents systematic differences in choices of which elements of the product environment people want to consider, differentiated by BMI category.

Our research may also have implications for creating study designs that ensure external validity of research findings. Many studies examining nutritional labeling start out in laboratory settings, with simple product choice environments. Frequently, participants will make choices between—or value—two products at a time. However, attention to product attributes may decrease as the number of alternatives to be considered increases [49], meaning that choices made in a simple choice set may yield attention to product attributes that would not be evaluated in a complex choice setting. Disparities found between field and laboratory experiments in the impact of front-of-package labels may be a result of varying the number of items that individuals consider [50,51,52]. Our results have implications for this observation in two ways: First, initial research on interventions meant to promote healthier diets may need to occur in richer choice environments, such as the environment we examine in this research, and in two other recent articles [36,53]. These experiments allow participants to interact with the array of products in a more realistic manner, which may mean choosing not to view some products that are available, including labels/information that may be available on those products. Second, we find that prompts may help redirect the attention of individuals who are more likely to choose to view less healthy products to a healthier set of products—a finding that could not be examined in a simple experimental choice setting.

The results related to the prompt show promise in terms of promoting the consideration of healthier alternatives. Health prompts have been shown to encourage healthier choices in laboratory and field settings [35,36,54,55], and may work in part by recruiting parts of the brain that are important in self-control and accelerating the consideration of health attributes in food choices [54,55]. While we studied a prompt delivered in an online environment, other prompts have been shown to be effective in physical supermarket settings [34,35]. Technological advances—either adopted personally by individuals, or by retailers—may also provide a means to deliver prompts in physical or online retail settings. Some of these capabilities are being developed in the context of mobile health (mHealth) applications [56].

Interestingly, participants across BMI categories responded similarly to the prompt in our study—a finding that differs from the conclusions of a study on health primes by Papies et al. [34]. There are a few differences in the studies that may explain the variation in results. In their study, the authors examined a subtle health prime on a recipe card provided to shoppers (i.e., the recipe was surrounded by words such as “healthy” and “good for your figure”, p. 599. [34]. The findings that overweight and obese individuals reduced unhealthy snack purchases (more than normal-weight individuals) applied only to those individuals who initially paid attention to the prime, whereas our study purposefully drew attention to the prompt message. Differences in the subtlety of the message may have led to different responses. For instance, the individuals in their study who paid attention to the health prime may have been more health-motivated, which other studies have shown to predict both attention to nutrition information and the healthiness of food choices and exercise behaviors [57,58,59,60].

This study does have some potential limitations. While hypothetical choices do not provide the same strength of evidence that real choices do, food choice is so frequent and deeply ingrained that it may be less subject to hypothetical biases than other product types [61]. In fact, recent research shows that food choices made in identical hypothetical and non-hypothetical settings exhibit similar patterns of choice, including the influence of hunger on choices [62]. The BMI characteristics of our sample indicate that we have fewer overweight/obese participants than the average in the US population. This may reflect two things: First, our sample is younger on average than the US adult population, which reduces the prevalence of high BMI [63]. Second, the use of self-reported measures of height and weight has been found in previous studies to lead to underestimates of BMI [64]. In addition, there is evidence that incorporating individuals’ perceived weight status—whether they believe that they are normal weight, overweight, or obese—in addition to BMI-based weight categorizations can shed additional light on food choice behavior [65,66,67,68,69]. Finally, samples drawn from Amazon’s Mechanical Turk (MTurk) are less representative of the US population than consumer panels maintained by survey companies (though more representative than in-person convenience samples) [70]. MTurk samples tend to be younger on average and have a different composition of race/ethnicity than consumer panels [71]. However, both consumer panels and MTurk samples over-represent urban populations [71].

The data for this study were collected during the early stages of the COVID-19 pandemic, which may have influenced multiple elements of the study: First, the sample in the study featured a higher percentage of males than females (only 36% of respondents were female). This may reflect the childcare burden faced by many women during the pandemic [72]. The pandemic also led many people to try shopping online for groceries for the first time. In our sample, 31% of participants reported shopping online for groceries for the first time during the previous month (only 25% had never shopped for groceries online). Finally, there is also evidence that the pandemic changed people’s eating behavior, with average nutritional quality decreasing with the onset of the pandemic [73].

Our findings suggest that even though BMI status predicts attention to product subsets that differ in nutritional quality, prompts hold promise in improving the quality of the sets that people consider, regardless of weight status. A study design that is specifically powered to examine relationships between BMI, attention to products and information and, ultimately, product choice, would shed important light on links between body weight status and directed attention towards health-ranked consideration sets, as well as the potential for prompt messages to shift attention and behavior towards healthier alternatives.

## Figures and Tables

**Table 1:** Demographic characteristics of the sample population ^a^.

	Control	Prompt
Female (%)	36%	35%
Age (Years)	37.2 (10.5)	36.6 (10.4)
Household Income (USD 10,000 s)	61.9 (28.9)	59.6 (28.5)
Education (Years)	15.9 (2.1)	15.8 (2.0)
BMI	25.5 (5.9)	25.5 (6.9)
N	253	500

^a^ Mean (standard error).

**Table 2:** Percentage of participants in each BMI category by condition.

Category	Normal Weight	Overweight	Obese
Control	42.8%	20.4%	36.8%
Prompt	43.0%	22.1%	34.9%

Notes: We omit individuals who did not submit height and/or weight data, preventing us from calculating BMI (n = 4: three in Control; one in Prompt).

**Table 3:** Linear probability model of choosing a consideration set that contains healthier options.

	(1)	(2)	(3)	(4)	(5)	(6)
Intercept	0.53 *** (0.02)	0.69 *** (0.03)	0.47 *** (0.03)	0.62 *** (0.11)	0.47 *** (0.03)	0.62 *** (0.11)
Overweight	0.02 (0.03)	0.01 (0.03)	0.02 (0.03)	0.01 (0.03)	0.04 (0.06)	0.02 (0.06)
Obese	−0.10 *** (0.03)	−0.10 *** (0.03)	−0.10 *** (0.03)	−0.10 *** (0.03)	−0.10 * (0.05)	−0.11 * (0.05)
Prompt			0.09 *** (0.03)	0.09 *** (0.03)	0.09 * (0.04)	0.09 * (0.04)
Overweight × Prompt					−0.03 (0.07)	−0.02 (0.07)
Obese × Prompt					0.01 (0.06)	0.02 (0.06)
Demographic Controls	No	Yes	No	Yes	No	Yes
*N*	749	739	749	739	749	739
Adj. R^2^	0.021	0.036	0.034	0.050	0.032	0.048

Notes: Estimate (standard error); * *p* < 0.05, *** *p* < 0.001. The models are linear probability models regressing the choice of a consideration set containing the healthier options (1 if yes, 0 if no) on the independent variables listed. Demographic control variables are female (1 if yes), age (numeric, in years), income (numeric in USD 1000s), and education (numeric, in years).

## Data Availability

Data are available from the authors upon request.

## Appendix A

**Table A1:** Bread products, nutritional and price information, and subsets in the experiment.

Bread Products	Cal.	Fat	Sodium	Fiber	Sugar	Price	Subset	Guiding Stars
Dave’s Killer Bread Good Seed	120	3	160	3	5	5.99	High	2
Dave’s Killer Bread Powerseed	100	2.5	135	4	1	5.99	High	3
Dave’s Killer Bread Thin Sliced Good Seed	70	1.5	115	3	2	5.99	High	2
Fiber Up 100% Whole Wheat	110	1.5	220	8	5	4.49	High	2
Fiber Up Multigrain	110	1.5	190	8	4	4.49	High	2
Oroweat Sandwich Thins 100% Whole Wheat	70	2	150	2	1.5	3.99	High	2
Pepperidge Farm 100% Whole Wheat	120	1	120	3	4	4.29	High	2
Pepperidge Farm Whole Grain 15 Grain	130	2.5	130	3	3	4.29	High	2
Thomas’ Light Multi-Grain English Muffin	50	1	85	4	0.5	3.49	High	2
Thomas’ 100% Whole Wheat English Muffin	60	1	115	1.5	0.5	3.49	High	3
Udi’s Omega Flax & Fiber	75	3	150	3	0.5	4.79	High	2
Pepperidge Farm Butter Bread	120	1	210	1	3	3.99	Low	0
Pepperidge Farm Hearty White	130	1	230	1	3	3.99	Low	0
Sara Lee Artesano Brioche	110	1.5	190	0.5	3	3.69	Low	0
Sara Lee Artesano Golden Wheat	100	1.5	180	1	3	3.69	Low	0
Thomas’ Bagels Blueberry	140	1	195	1	4.5	4.69	Low	0
Thomas’ Bagels Cinnamon Swirl	140	1	195	1.5	5.5	4.69	Low	0
Thomas’ Bagels Plain	135	1	225	1	3	4.69	Low	0
Thomas’ English Muffin Cinnamon Raisin	150	0.5	180	2	4	4.49	Low	0
Thomas’ English Muffin Original	75	0.5	120	0.5	2	4.49	Low	0
Udi’s Gluten-Free Plain Bagel	160	5	295	1.5	0	4.98	Low	0
Udi’s Gluten-Free White	70	2	135	0.5	1.5	4.98	Low	0
Dave’s Killer Bread White	110	2	180	2	2	5.99	Medium	1
Oroweat Whole Grains 12 Grain	100	2	160	3	2	3.99	Medium	1
Oroweat Whole Grains Oatnut	110	2	135	2	3	3.99	Medium	1
Sara Lee 100% Whole Wheat	60	1	120	2	1	3.99	Medium	1
Sara Lee Butter Bread	70	0.5	110	0	1	3.99	Medium	1
Sara Lee Delightful 45 Calories 100% Whole Wheat	45	0.5	100	1.5	1	3.99	Medium	1
Sara Lee Delightful 45 Calories Multi-Grain	45	0.5	85	1.5	1	3.99	Medium	1
Sara Lee Honey Wheat	70	1	120	0.5	1	3.99	Medium	1
Thomas’ Bagel 100% Whole Wheat	125	0.5	125	3.5	3.5	4.69	Medium	1
Thomas’ Bagel Thins Plain	55	0.5	105	2	1	3.99	Medium	1
Udi’s Gluten-Free Millet-Chia	75	2	150	2.5	0.5	4.79	Medium	1

Note: Nutritional information was provided on a standardized per-serving basis. Note that while the table presents the subset in which each product was included for participants who chose to see a subset, participants could also choose to view all available products in a particular category. We have also categorized product subsets by relative nutritional quality in this table rather than presenting the text used in the experiment, given the length of the descriptors. The text used in the experiment was (1) “Breads such as Dave’s Killer Powerseed, Fiber Up 100% Whole Wheat, Pepperidge Farm 15 Grain” (=High in this table); (2) “Breads such as Sara Lee 100% Whole Wheat, Thomas’ Bagel Thins Plain, Oroweat Oatnut” (=Medium in this table); and (3) “Breads such as Sara Lee Artesano Golden Wheat, Pepperidge Farm Hearty White, Thomas’ Plain Bagels” (=Low in this table).

**Table A2:** Cereal products, nutritional and price information, and subsets in the experiment.

Cereals	Cal.	Fat	Sodium	Fiber	Sugar	Price	Subset	Guiding Stars
All-Bran Buds	120	2	95	12	9	4.49	High	2
Cheerios	140	2.5	190	4	2	3.49	High	2
Fiber One Original	90	1.5	140	14	0	4.29	High	3
Frosted Mini-Wheats Original	140	1	10	4	6	2.88	High	2
Grape-Nuts	138	1	193	5	3	3.12	High	3
Great Grains Raisins Dates Pecans	200	1	150	5	13	3.18	High	2
Kashi Berry Fruitful	125	1	0	4	6	3.97	High	2
Multi-Grain Cheerios	150	2	150	4	8	3.49	High	2
Shredded Wheat	140	1	0	5	0	2.88	High	3
Wheat Chex	142	1	231	5	4	3.79	High	2
Wheaties	144	0.5	267	4	6	4.29	High	2
Apple Jacks	150	1.5	210	2	13	3.68	Low	0
Cap’n Crunch’s Crunch Berries	150	2	270	0.5	16	2.79	Low	0
Cookie Crisp	155	3	170	2	13	3.49	Low	0
Corn Pops	150	0.5	140	0	12	3.68	Low	0
Froot Loops	152	1.5	210	4	14	3.29	Low	0
Frosted Flakes	140	0	200	0.5	14	3.29	Low	0
Fruity Pebbles	155	2	210	0	13	2.99	Low	0
Honey Comb	160	1	190	1	13	3.19	Low	0
Lucky Charms	155	2	255	2	13	3.4	Low	0
Reese’s Puffs	170	4.5	210	2	12	2.99	Low	0
Trix	160	2	180	1	12	3.46	Low	0
Crispix	150	0	260	0	5	3.68	Medium	1
Corn Flakes	150	0	300	1	4	3.78	Medium	1
Golden Grahams	160	1	300	2	12	3.49	Medium	1
Oatmeal Squares	150	2	136	4	6	4.48	Medium	1
Special K Banana	160	2.5	230	3	9	3.19	Medium	1
Special K Blueberry with Lemon Clusters	150	1	260	3	12	3.19	Medium	1
Special K Cinnamon Brown Sugar Crunch Protein	160	1	230	4	12	3.19	Medium	1
Special K Cinnamon Pecan	160	2.5	280	3	10	3.19	Medium	1
Special K Original Protein	142	1	176	3	5	3.19	Medium	1
Special K Raspberry	150	0.5	230	3	12	3.19	Medium	1
Special K Red Berries	140	0.5	250	3	11	3.19	Medium	1

Note: Nutritional information was provided on a standardized per-serving basis. Note that while the table presents the subset in which each product was included for participants who chose to see a subset, participants could also choose to view all available products in a particular category. We have also categorized product subsets by relative nutritional quality in this table rather than presenting the text used in the experiment, given the length of the descriptors. The text used in the experiment was (1) “Cereals such as Cheerios, Wheat Chex, Grape Nuts” (=High in this table); (2) “Cereals such as Corn Flakes, Crispix, Special K” (=Medium in this table); and (3) “Cereals such as Frosted Flakes, Froot Loops, Reese’s Puffs” (=Low in this table).

**Table A3:** Cracker products, nutritional and price information, and subsets in the experiment.

Crackers	Cal.	Fat	Sodium	Fiber	Sugar	Price	Subset	Guiding Stars
Blue Diamond Artisan Nut Thins Flax Seeds	130	3.5	135	2	0	3.99	High	2
Farmhouse Cheddar Almond Flour	150	8	270	1	0.5	5.69	High	2
Farmhouse Sprouted Seed Original	140	8	210	3	0	5.69	High	2
Pepperidge Farm Goldfish Baked with Whole Grain	140	5	240	2	0	2.49	High	2
Triscuit Balsamic Vinegar & Basil	130	4	130	3	0.5	3.38	High	2
Triscuit Cracked Pepper and Olive Oil	130	4.5	150	3	0	3.38	High	2
Triscuit Original	130	4.5	170	4	0	3.38	High	2
Triscuit Reduced Fat Crackers	120	2.5	160	4	0	3.38	High	2
Wasa Light Rye	67	0	117	7	0	3.49	High	3
Wasa Multi-Grain	75	0	139	6	0	3.49	High	2
Wasa Whole Grain	69	0	115	7	0	3.49	High	2
Cheez-It Hot & Spicy	150	8	220	1	0	3.69	Low	0
Cheez-It Original	150	8	230	1	0	3.69	Low	0
Cheez-It Pepper Jack	150	7	270	1	0.5	3.69	Low	0
Cheez-It White Cheddar	150	7	210	1	0	3.69	Low	0
Keebler Cheese & Peanut Butter	145	7	240	0.5	3	3.59	Low	0
Keebler Club & Cheddar	145	7	240	0.5	4	3.59	Low	0
Keebler Original Club	150	6.5	268	0	2	2.99	Low	0
Keebler Town House Flipside Pretzel Original	140	7	380	0	2	4.49	Low	0
Keebler Town House Original	150	9.5	280	0	2	4.49	Low	0
Keebler Town House Sea Salt Pita Crackers	140	5	270	0	0.5	4.49	Low	0
Nabisco Ritz Original Classic	150	8.5	244	0	2	3.38	Low	0
Crunchmaster Multi-Grain Sea Salt	120	3	140	3	1	3.99	Medium	1
Crunchmaster Multi-Seed Original	140	5	110	2	0	3.99	Medium	1
Crunchmaster Multi-Seed Roasted Garlic	140	5.5	135	2	0	3.99	Medium	1
Crunchmaster Multi-Seed Rosemary & Olive Oil	140	5	90	2	0	3.99	Medium	1
Good Thins: The Beet One—Balsamic Vinegar & Sea Salt	130	4	160	2	3	4.38	Medium	1
Good Thins: The Cheese One—White Cheddar	130	4	180	2	2	4.38	Medium	1
Good Thins: The Potato One—Spinach & Garlic	130	4	190	3	1	3.38	Medium	1
Good Thins: The Rice One—Simply Salt	130	1.5	85	0	0	3.38	Medium	1
Good Thins: The Rice One—Veggie Blend	120	1.5	90	1	2	3.38	Medium	1
Nabisco Wheat Thins Multigrain	130	4	190	2	3	3.38	Medium	1
Pepperidge Farm Goldfish Cheddar	140	5	250	0.5	0	2.49	Medium	1

Note: Nutritional information was provided on a standardized per-serving basis. Note that while the table presents the subset in which each product was included for participants who chose to see a subset, participants could also choose to view all available products in a particular category. We have also categorized product subsets by relative nutritional quality in this table rather than presenting the text used in the experiment, given the length of the descriptors. The text used in the experiment was (1) “Crackers such as Wasa, Triscuit, Simple Mill Crackers” (=High in this table); (2) “Crackers such as Wheat Thins, Good Thins, Crunchmaster” (=Medium in this table); and (3) “Crackers such as Cheez-It, Ritz, Club Original” (=Low in this table).

## Appendix B

**Table A4:** Ordinal logistic regression model of choosing a consideration set that contains healthier options. Results represent odds ratios and 95% confidence intervals.

	(1)	(2)	(3)	(4)	(5)	(6)
Overweight	1.13 (0.80, 1.59)	1.05 (0.74, 1.59)	1.11 (0.79, 1.56)	1.03 (0.73, 1.47)	1.24 (0.68, 2.28)	1.11 (0.60, 2.05)
Obese	0.59 (0.44, 0.79)	0.59 (0.43, 0.79)	0.59 (0.44, 0.79)	0.59 (0.44, 0.80)	0.58 (0.35, 0.95)	0.55 (0.33, 0.91)
Prompt			1.60 (1.22, 2.11)	1.65 (1.25, 2.18)	1.64 (1.08, 2.50)	1.62 (1.06, 2.48)
Overweight × Prompt					0.85 (0.41, 1.77)	0.91 (0.43, 1.89)
Obese × Prompt					1.03 (0.56, 1.91)	1.11 (0.60, 2.08)
Demographic Controls?	No	Yes	No	Yes	No	Yes
N	749	739	749	739	749	739
AIC	2039.1	2004.2	2029.7	1993.5	2033.4	1997.2

## References

[1] Afshin A., Forouzanfar M.H., Reitsma M.B., Sur P., Estep K., Lee A., Marczak L., Mokdad A.H., Moradi-Lakeh M., GBD 2015 Obesity Collaborators (2017). Health effects of overweight and obesity in 195 countries over 25 years. N. Engl. J. Med..

[2] Harper S., Riddell C.A., King N.B. (2020). Declining life expectancy in the United States: Missing the trees for the forest. Annu. Rev. Public Health.

[3] Mehta N.K., Abrams L.R., Myrskylä M. (2020). US life expectancy stalls due to cardiovascular disease, not drug deaths. Proc. Natl. Acad. Sci. USA.

[4] Preston S.H., Vierboom Y., Stokes A. (2018). The role of obesity in exceptionally slow US mortality improvement. Proc. Natl. Acad. Sci. USA.

[5] Centers for Disease Control and Prevention (CDC) Adult Obesity Facts. https://www.cdc.gov/obesity/data/adult.html.

[6] Cawley J. (2004). The impact of obesity on wages. J. Hum. Resour..

[7] Finkelstein E., Fiebelkorn L.C., Wang G. (2005). The costs of obesity among full-time employees. Am. J. Health Promot..

[8] Finkelstein E.A., Trogdon J.G., Cohen J.W., Dietz W. (2009). Annual medical spending attributable to obesity: Payer and service-specific estimates. Health Aff..

[9] Hales C.M., Fryar C.D., Carroll M.D., Freedman D.S., Ogden C.L. (2018). Trends in obesity and severe obesity prevalence in US youth and adults by sex and age, 2007–2008 to 2015–2016. JAMA.

[10] Pontzer H., Raichlen D.A., Wood B.M., Mabulla A.Z.P., Racette S., Marlowe F.W. (2012). Hunter-gatherer energetics and human obesity. PLoS ONE.

[11] Urlacher S.S., Snodgrass J.J., Dugas L.R., Sugiyama L.S., Liebert M.A., Joyce C.J., Pontzer H. (2019). Constraint and trade-offs regulate energy expenditure during childhood. Sci. Adv..

[12] Urlacher S.S., Snodgrass J.J., Dugas L.R., Madimenos F.C., Sugiyama L.S., Liebert M.A., Joyce C.J., Terán E., Pontzer H. (2021). Childhood daily energy expenditure does not decrease with market integration and is not related to adiposity in Amazonia. J. Nutr..

[13] Hall K.D., Ayuketah A., Brychta R., Cai H., Cassimatis T., Chen K., Chung S.T., Costa E., Courville A., Darcey V. (2019). Ultra-processed diets cause excess calorie intake and weight gain: An inpatient randomized controlled trial of ad libitum food intake. Cell Metab..

[14] Cummins S., Cockerham W.C., Dingwall R., Quah S. (2014). Food deserts. The Wiley Blackwell Encyclopedia of Health, Illness, Behavior, and Society.

[15] Chen D., Jaenicke E.C., Volpe R.J. (2016). Food environments and obesity: Household diet expenditure versus food deserts. Am. J. Public Health.

[16] Ver Ploeg M., Breneman V., Farrigan T., Hamrick K., Hopkins D., Kaufman P., Lin B.-H., Nord M., Smith T.A., Williams R. Access to Affordable and Nutritious Food-Measuring and Understanding Food Deserts and Their Consequences: Report to Congress. https://www.ers.usda.gov/publications/pub-details/?pubid=42729.

[17] Cummins S., Flint E., Matthews S. (2014). New neighborhood grocery store increased awareness of food access but did not alter dietary habits or obesity. Health Aff..

[18] Dubowitz T., Ghosh-Dastidar M., Cohen D.A., Beckman R., Steiner E.D., Hunter G.P., Flórez K., Huang C., Vaughan C.A., Sloan J.C. (2015). Diet and perceptions change with supermarket introduction in a food desert, but not because of supermarket use. Health Aff..

[19] Ghosh-Dastidar M., Hunter G., Collins R.L., Zenk S.N., Cummins S., Beckman R., Nugroho A.K., Sloan J.C., Wagner L., Dubowitz T. (2017). Does opening a supermarket in a food desert change the food environment?. Health Place.

[20] Allcott H., Diamond R., Dubé J.-P., Handbury J., Rahkovsky I., Schnell M. (2019). Food deserts and the causes of nutritional inequality. Q. J. Econ..

[21] Botti S., Iyengar S.S. (2006). The dark side of choice: When choice impairs social welfare. J. Public Policy Mark..

[22] Zizzo D.J., Parravano M., Nakamura R., Forwood S., Suhrcke M. (2021). The impact of taxation and signposting on diet: An online field study with breakfast cereals and soft drinks. Exp. Econ..

[23] Hauser J.R., Wernerfelt B. (1990). An evaluation cost model of consideration sets. J. Consum. Res..

[24] Roberts J.H., Lattin J.M. (1991). Development and testing of a model of consideration set composition. J. Mark. Res..

[25] Honka E., Hortaçsu A., Wildenbeest M., Dubé J.-P., Rossi P.E. (2019). Chapter 4—Empirical search and consideration sets. Handbook of the Economics of Marketing.

[26] Machín L., Curutchet M.R., Gugliucci V., Vitola A., Otterbring T., de Alcantara M., Ares G. (2020). The habitual nature of food purchases at the supermarket: Implications for policy making. Appetite.

[27] Caplin A., Dean M., Leahy J. (2019). Rational inattention, optimal consideration sets, and stochastic choice. Rev. Econ. Stud..

[28] Masatlioglu Y., Nakajima D., Ozbay E.Y. (2012). Revealed attention. Am. Econ. Rev..

[29] Haws K.L., Reczek R.W., Sample K.L. (2016). Healthy diets make empty wallets: The healthy = expensive intuition. J. Consum. Res..

[30] Jo J., Lusk J.L. (2018). If it’s healthy, it’s tasty and expensive: Effects of nutritional labels on price and taste expectations. Food Qual. Prefer..

[31] Lusk J.L. (2019). Consumer beliefs about healthy foods and diets. PLoS ONE.

[32] Nansel T.R., Lipsky L.M., Eisenberg M.H., Liu A., Mehta S.N., Laffel L.M. (2016). Can families eat better without spending more? Improving diet quality does not increase diet cost in a randomized clinical trial among youth with type 1 diabetes and their parents. J. Acad. Nutr. Diet..

[33] Elbel B., Kersh R., Brescoll V.L., Dixon L.B. (2009). Calorie labeling and food choices: A first look at the effects on low-income people in New York City: Calorie information on menus appears to increase awareness of calorie content, but not necessarily the number of calories people purchase. Health Aff..

[34] Papies E.K., Potjes I., Keesman M., Schwinghammer S., van Koningsbruggen G. (2013). Using health primes to reduce unhealthy snack purchases among overweight consumers in a grocery store. Int. J. Obes..

[35] Gustafson C.R., Kent R., Prate M.R. (2018). Retail-based healthy food point-of-decision prompts (PDPs) increase healthy food choices in a rural, low-income, minority community. PLoS ONE.

[36] Arslain K., Gustafson C.R., Rose D.J. (2020). Point-of-decision prompts increase dietary fiber content of consumers’ food choices in an online grocery shopping simulation. Nutrients.

[37] Zhao A.W., McGowan C.C., Zenk S.N., Kershaw K.N. (2020). Associations of the consumer food environment with eating behaviours and BMI. Public Health Nutr..

[38] Bello M., Abdulai A. (2016). Impact of ex-ante hypothetical bias mitigation methods on attribute non-attendance in choice experiments. Am. J. Agric. Econ..

[39] Bunn A., Korpela M. (2018). R: A Language and Environment for Statistical Computing.

[40] McKenna R.M., Langellier B.A., Alcalá H.E., Roby D.H., Grande D.T., Ortega A.N. (2018). The affordable care act attenuates financial strain according to poverty level. Inq. J. Health Care Organ. Provis. Financ..

[41] Dubowitz T., Ncube C., Leuschner K., Tharp-Gilliam S. (2015). A natural experiment opportunity in two low-income urban food desert communities: Research design, community engagement methods, and baseline results. Health Educ. Behav..

[42] Vaughan C.A., Cohen D.A., Ghosh-Dastidar M., Hunter G.P., Dubowitz T. (2016). Where do food desert residents buy most of their junk food? Supermarkets. Public Health Nutr..

[43] Hut S. (2020). Determinants of dietary choice in the US: Evidence from consumer migration. J. Health Econ..

[44] Aviram-Friedman R., Astbury N., Ochner C.N., Contento I., Geliebter A. (2018). Neurobiological evidence for attention bias to food, emotional dysregulation, disinhibition and deficient somatosensory awareness in obesity with binge eating disorder. Physiol. Behav..

[45] Nijs I.M.T., Franken I.H.A. (2012). Attentional processing of food cues in overweight and obese individuals. Curr. Obes. Rep..

[46] Werthmann J., Roefs A., Nederkoorn C., Mogg K., Bradley B.P., Jansen A. (2011). Can(not) take my eyes off it: Attention bias for food in overweight participants. Health Psychol..

[47] Just D.R., Wansink B. (2013). One man’s tall is another man’s small: How the framing of portion size influences food choice. Health Econ..

[48] Werthmann J., Jansen A., Roefs A. (2014). Worry or craving? A selective review of evidence for food-related attention biases in obese individuals, eating-disorder patients, restrained eaters and healthy samples. Proc. Nutr. Soc..

[49] Meißner M., Oppewal H., Huber J. (2020). Surprising adaptivity to set size changes in multi-attribute repeated choice tasks. J. Bus. Res..

[50] Dubois P., Albuquerque P., Allais O., Bonnet C., Bertail P., Combris P., Lahlou S., Rigal N., Ruffieux B., Chandon P. (2020). Effects of front-of-pack labels on the nutritional quality of supermarket food purchases: Evidence from a large-scale randomized controlled trial. J. Acad. Mark. Sci..

[51] Gustafson C.R., Zeballos E. (2018). The effect of ingredient-specific calorie information on calories ordered. Prev. Med. Rep..

[52] Streletskaya N.A., Amatyakul W., Rusmevichientong P., Kaiser H.M., Liaukonyte J. (2015). Menu-labeling formats and their impact on dietary quality. Agribusiness.

[53] Arslain K., Gustafson C.R., Rose D.J. (2021). The effect of health prompts on product consideration, attention to information, and choice in large, online product assortments: The case of fiber. Food Qual. Prefer..

[54] Hare T.A., Malmaud J., Rangel A. (2011). Focusing attention on the health aspects of foods changes value signals in vmPFC and improves dietary choice. J. Neurosci..

[55] Lim S.-L., Penrod M.T., Ha O.-R., Bruce J.M., Bruce A.S. (2018). Calorie labeling promotes dietary self-control by shifting the temporal dynamics of health- and taste-attribute integration in overweight individuals. Psychol. Sci..

[56] Silva B.M., Rodrigues J.J., de la Torre Díez I., López-Coronado M., Saleem K. (2015). Mobile-health: A review of current state in 2015. J. Biomed. Inform..

[57] Moorman C. (1996). A quasi experiment to assess the consumer and informational determinants of nutrition information processing activities: The case of the nutrition labeling and education act. J. Public Policy Mark..

[58] Visschers V., Hess R., Siegrist M. (2010). Health motivation and product design determine consumers’ visual attention to nutrition information on food products. Public Health Nutr..

[59] Turner M.M., Skubisz C., Pandya S.P., Silverman M., Austin L. (2014). Predicting visual attention to nutrition information on food products: The influence of motivation and ability. J. Health Commun..

[60] Naughton P., McCarthy S.N., McCarthy M.B. (2015). The creation of a healthy eating motivation score and its association with food choice and physical activity in a cross-sectional sample of Irish adults. Int. J. Behav. Nutr. Phys. Act..

[61] Rangel A. (2013). Regulation of dietary choice by the decision-making circuitry. Nat. Neurosci..

[62] De-Magistris T., López-Galán B., Ballco P. (2021). Do virtual reality experiments replicate projection bias phenomena? Examining the external validity of a virtual supermarket. J. Agric. Econ..

[63] Fryar C.D., Carroll M.D., Afful J. (2020). Prevalence of overweight, obesity, and extreme obesity among adults aged 20 and over: United States, 1960–1962 through 2017–2018.

[64] Gorber S.C., Tremblay M., Moher D., Gorber B. (2007). A comparison of direct vs. self-report measures for assessing height, weight and body mass index: A systematic review. Obes. Rev..

[65] Mbarushimana J.-C., Gustafson C., Gitungwa H., Zeballos E. (2021). The relationship between bodyweight status and weight perception explains differences in calories ordered in a food choice exercise. Nutrients.

[66] Wharton C.M., Adams T., Hampl J. (2008). Weight loss practices and body weight perceptions among US college students. J. Am. Coll. Health.

[67] Lemon S.C., Rosal M.C., Zapka J., Borg A., Andersen V. (2009). Contributions of weight perceptions to weight loss attempts: Differences by body mass index and gender. Body Image.

[68] Chen H.-Y., Lemon S.C., Pagoto S.L., Barton B.A., Lapane K.L., Goldberg R.J. (2014). Personal and parental weight misperception and self-reported attempted weight loss in US children and adolescents, national health and nutrition examination survey, 2007–2008 and 2009–2010. Prev. Chronic Dis..

[69] Yaemsiri S., Slining M.M., Agarwal S.K. (2010). Perceived weight status, overweight diagnosis, and weight control among US adults: The NHANES 2003–2008 study. Int. J. Obes..

[70] Berinsky A.J., Huber G.A., Lenz G.S. (2012). Evaluating online labor markets for experimental research: Amazon.com’s mechanical Turk. Political Anal..

[71] Huff C., Tingley D. (2015). “Who are these people?” Evaluating the demographic characteristics and political preferences of MTurk survey respondents. Res. Politics.

[72] Sevilla A., Smith S. (2020). Baby steps: The gender division of childcare during the COVID-19 pandemic. Oxf. Rev. Econ. Policy.

[73] Marty L., de Lauzon-Guillain B., Labesse M., Nicklaus S. (2021). Food choice motives and the nutritional quality of diet during the COVID-19 lockdown in France. Appetite.

